# Identification of tumor antigens and immune subtypes of pancreatic adenocarcinoma for mRNA vaccine development

**DOI:** 10.1186/s12943-021-01310-0

**Published:** 2021-03-01

**Authors:** Xing Huang, Gang Zhang, Tianyu Tang, Tingbo Liang

**Affiliations:** 1 Department of Hepatobiliary and Pancreatic Surgery, the First Affiliated Hospital, School of Medicine, Zhejiang University, Zhejiang, 310003 Hangzhou China; 2 Zhejiang Provincial Key Laboratory of Pancreatic Disease, the First Affiliated Hospital, School of Medicine, Zhejiang University, Zhejiang, 310003 Hangzhou China; 3 Innovation Center for the Study of Pancreatic Diseases, Zhejiang Province, Zhejiang, 310003 Hangzhou China; 4 Zhejiang University Cancer Center, Zhejiang, 310003 Hangzhou China; 5 Research Center for Healthcare Data Science, Zhejiang Lab, Zhejiang, 310003 Hangzhou China

**Keywords:** mRNA vaccine, Immunotype, Pancreatic adenocarcinoma, Tumor immune microenvironment, Immune landscape

## Abstract

**Background:**

Although mRNA vaccines have been effective against multiple cancers, their efficacy against pancreatic adenocarcinoma (PAAD) remains undefined. Accumulating evidence suggests that immunotyping can indicate the comprehensive immune status in tumors and their immune microenvironment, which is closely associated with therapeutic response and vaccination potential. The aim of this study was to identify potent antigens in PAAD for mRNA vaccine development, and further distinguish immune subtypes of PAAD to construct an immune landscape for selecting suitable patients for vaccination.

**Methods:**

Gene expression profiles and clinical information of 239 PAAD datasets were extracted from ICGC, and RNA-Seq data of 103 samples were retrieved from TCGA. GEPIA was used to calculate differential expression levels and prognostic indices, cBioPortal program was used to compare genetic alterations, and TIMER was used to explore correlation between genes and immune infiltrating cells. Consensus cluster was used for consistency matrix construction and data clustering, DAVID was used for functional annotation, and graph learning-based dimensional reduction was used to depict immune landscape.

**Results:**

Six overexpressed and mutated tumor antigens associated with poor prognosis and infiltration of antigen presenting cells were identified in PAAD, including ADAM9, EFNB2, MET, TMOD3, TPX2, and WNT7A. Furthermore, five immune subtypes (IS1-IS5) and nine immune gene modules of PAAD were identified that were consistent in both patient cohorts. The immune subtypes showed distinct molecular, cellular and clinical characteristics. IS1 and IS2 exhibited immune-activated phenotypes and correlated to better survival compared to the other subtypes. IS4 and IS5 tumors were immunologically cold and associated with higher tumor mutation burden. Immunogenic cell death modulators, immune checkpoints, and CA125 and CA199, were also differentially expressed among the five immune subtypes. Finally, the immune landscape of PAAD showed a high degree of heterogeneity between individual patients.

**Conclusions:**

ADAM9, EFNB2, MET, TMOD3, TPX2, and WNT7A are potent antigens for developing anti-PAAD mRNA vaccine, and patients with IS4 and IS5 tumors are suitable for vaccination.

## Background

Pancreatic adenocarcinoma (PAAD) is a lethal malignancy with a 5-year survival rate of only ~ 10% [1–3]. It is the 7th leading cause of cancer-related mortality, and accounted for 4.5% of cancer deaths worldwide in 2018 [4]. Since localized pancreatic cancer is largely asymptomatic in the early stages, > 80% of the patients are typically diagnosed in the advanced or metastatic phase, rendering surgical intervention impossible [5–7]. Furthermore, the patients that undergo complete tumor resection often present with local or distant recurrence within 2 years of operation [7]. Other therapeutic strategies, such as combination chemotherapy, molecular targeted drugs and immune checkpoint inhibitors also have limited efficacy due to intrinsic chemo- and immune- resistance [8–10]. Recently, tumor vaccines targeting the ganglioside GD2 and CA-199/KLH have been successful in mitigating PAAD progression [11, 12]. Although these vaccines provided a survival benefit of only several months, the results are encouraging enough to explore the potential of PAAD-related vaccines.

Prototypical cancer vaccines consist of a tumor antigen with/without an adjuvant, which reprograms the immune system to recognize and eliminate cancer cells. They have the advantages of relative non-toxicity, minimal non-specific effects, broad therapeutic window and induction of persistent immunological memory [13, 14]. Therefore, cancer vaccines can overcome the drug resistance, adverse reactions, limited therapeutic efficacy and high costs associated with standard chemo- and immunotherapies [13]. Based on the antigen form, cancer vaccines are classified into the peptide, tumor cell, dendritic cell, DNA and RNA types [15, 16]. However, the first four types have several disadvantages that limit their clinical potential. For instance, development of personalized peptide vaccines require genetic analysis of the tumor, which is not possible for inoperable pancreatic cancer and may postpone the treatment [14, 15]. In addition, development of DC vaccines is time and resource consuming, and although they can trigger Th and CTL cells responses, the therapeutic efficacy is poor [15]. Tumor cell vaccines showed promising outcomes in preclinical models but failed in clinical trials [15]. Finally, DNA sequences can integrate into the tumor cell genome and cause insertional mutations, eventually neutralizing the therapeutic effect [17, 18]. In contrast, messenger RNA (mRNA) does not pose any risk of insertional mutagenesis since it cannot integrate into the genome. It is also degraded by cellular RNases and therefore has a short and regulatable half-life in vivo, indicating good safety profile [16, 18, 19]. Furthermore, advances in sequence modification and carrier strategies have increased the in vivo stability, cellular uptake and expression of mRNA molecules [20–22]. In the context of clinical applications, mRNA vaccines can be manufactured in a cost-effective manner due to development of rapid in vitro transcription reactions. Finally, mRNA sequences can be designed and modified to encode any pathological antigen [23], which is highly significant during a pandemic or for individualized therapies [18]. Therefore, mRNA vaccines are highly suitable for targeting tumor-specific antigens [14], and multiple preclinical and clinical trials have been initiated [18, 19]. For example, Sebastian et al. reported that the RNActive® vaccine CV9201 induced antigen-specific immune response in 65% (30/46) of stage IIIB/IV non-small cell lung cancer patients, and achieved stable disease in 31% evaluable patients and treatment-free survival of 5 stage IV patients for more than 1 year [17]. Likewise, Kübler et al. demonstrated that CV9103 triggered an immune response in advanced castration-resistant prostate cancer patients and prolonged overall survival (OS) in a phase I/IIa study, and remained well-tolerated and immunogenic [24]. However, no mRNA vaccine against pancreatic cancer antigens has been developed so far, and no patient sub-population suitable for vaccination has been identified.

The aim of our study was to explore novel PAAD antigens for developing mRNA vaccines, and map the immune landscape of PAAD to select suitable patients for vaccination. We identified 6 candidates associated with poor survival and antigen presenting cell (APC) infiltration from the pool of overexpressed and mutated genes in PAAD. Based on the clustering of immune-related genes, we then defined 5 robust immune subtypes and 9 functional modules of PAAD, and validated them in an independent cohort. Each immune subtype corresponded to distinct clinical, molecular and cellular characteristics. Finally, the immune landscape of PAAD was defined by analyzing the distribution of the relevant gene signatures among individual patients. Our findings point to a complex tumor immune microenvironment (TIME) in each PAAD patient, and provide the theoretical basis for developing mRNA vaccines and selecting suitable patients for vaccination.

## Methods

### Data extraction

The normalized gene expression and clinical follow-up data of 239 PAAD patients, and RNA-seq data of 103 patients were retrieved from International Cancer Genome Consortium (ICGC, https://www.icgc-argo.org) and The Cancer Genome Atlas (TCGA, https://www.cancer.gov/tcga) respectively through GDC API. A total of 2006 immune-related genes were extracted from both the discovery and validation cohorts, and included single immune cell-specific, co-stimulating and co-suppressing molecules-related, cytokines and cytokines receptors-related, antigen processing and presenting-related, and other immune related genes.

### Data preprocessing

To process the ICGC data, the microarray probes with null gene test value were first removed and the remaining probes were mapped to human genes. A total of 239 immune cell-related genes were retained from 1982 expressed genes. TCGA tumor samples lacking clinical information and the normal tissue data were first excluded, followed by genes with 0 FPKM in > 50% of the samples. Finally, 103 immune cell-related genes with log2(FPKM + 1) fold-change were included for the subsequent analysis.

### GEPIA analysis

The raw RNA-Seq data downloaded from TCGA and ICGC databases were recomputed using UCSC Xena to avoid data imbalance and inefficient differential analyses. Differential gene expression and patient survival data were integrated using Gene Expression Profiling Interactive Analysis [25] (GEPIA, http://gepia2.cancer-pku.cn, version 2). ANOVA was used to identify the differentially expressed genes with |log2FC| values > 1 and q values < 0.01. OS and relapse-free survival (RFS) were evaluated using the Kaplan-Meier method with a 50% (Median) cutoff, and compared by the log rank test. The cox proportional hazards regression model was applied to calculate the hazard ratio. The parameter setting was consistent in each analysis without adjustment for any *p* value. *P*-values < 0.05 were considered statistically significant.

### cBioPortal analysis

The cBio Cancer Genomics Portal [26] (cBioPortal, http://www.cbioportal.org) was used to integrate the raw RNA-seq data from TCGA, ICGC and other databases, and compare genetic alterations in PAAD. *P*-values < 0.05 were considered statistically significant.

### TIMER analysis

Tumor Immune Estimation Resource [27] (TIMER, https://cistrome.shinyapps.io/timer/) was used to analyze and visualize the association between abundance of tumor immune infiltrating cells (TIICs) and PAAD-related genes through analytical modules for gene expression, somatic mutations, clinical outcomes and somatic copy number alteration. Purity Adjustment was selected using Spearman’s correlation analysis. *P*-values < 0.05 were considered statistically significant.

### Discovery and validation of the immune subtypes

The 19,972 immune-related genes were clustered on the basis of their expression profiles, and a consistency matrix was constructed to identify corresponding immune subtypes and gene modules. The partition around medoids algorithm using the “1-Pearson correlation” distance metric was applied, and 500 bootstraps were performed each involving 80% patients in the discovery cohort. Cluster sets varied from 2 to 10, and the optimal partition was defined by evaluating the consensus matrix and the consensus cumulative distribution function. The immune subtypes were then validated in an independent TCGA cohort with the same settings. The consistency of immune subtypes between the discovery and validation cohorts were quantified by calculating intra-group proportion and Pearson correlation in the centroids of gene module scores.

### Prognostic evaluation of immune subtypes

The prognostic values of the immune subtypes were evaluated using log-rank test, and univariate and multivariate cox regression, with stage and grade as covariates, and OS and RPS as the endpoints. ANOVA was used to determine the association of immune subtypes with different immune-related molecular and cellular characteristics. The most frequently mutated genes were screened using the chi-square test. DAVID program was used to functionally annotate each gene module through gene ontology analysis. Single-sample GSEA (ssGSEA) was used to calculate immune enrichment scores for each sample, which is the measure of genes that are coordinately up- or down-regulated within a sample.

### Construction of immune landscape

The graph learning-based dimensionality reduction analysis was performed to visualize the distribution of immune subtypes across individual patients using the reduce Dimension function of Monocle package with a Gaussian distribution. The maximum number of components was set to 4, and the discriminative dimensionality reduction with trees was used. Finally, the immune landscape was visualized with the function plot cell trajectory with color-coded immune subtypes.

## Results

### Identification of potential antigens of PAAD

**Fig. 1:**
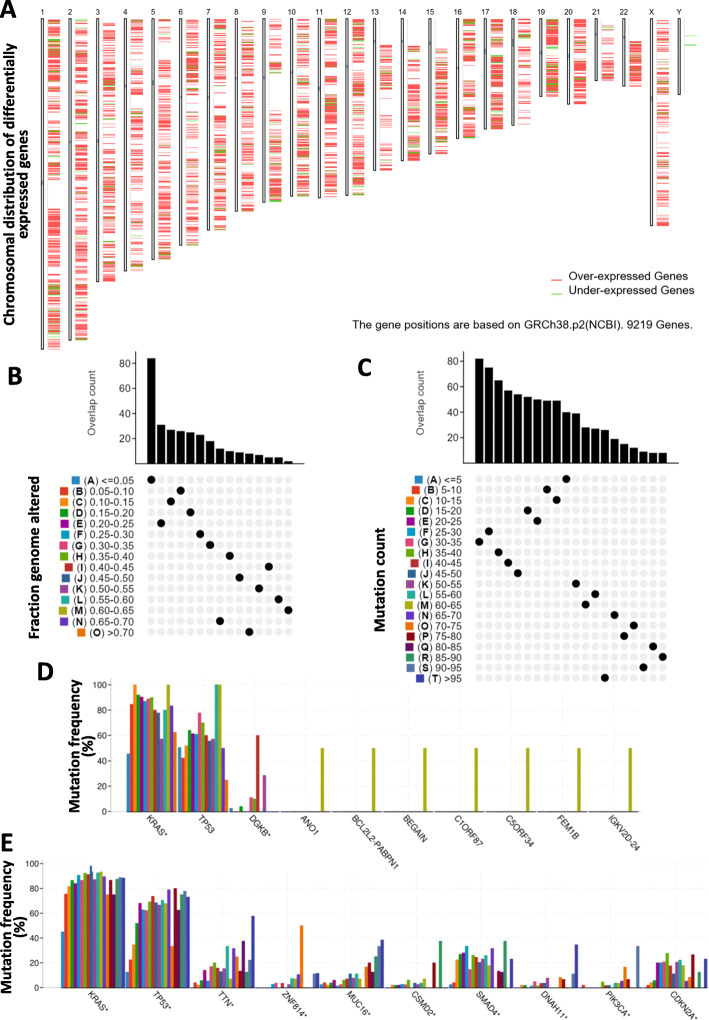
Identification of potential tumor antigens of PAAD. **a** Identification of potential tumor-associated antigens of PAAD. Chromosomal distribution of up- and down-regulated genes in PAAD as indicated. **b**-**e** Identification of potential tumor-specific antigens of PAAD. Samples overlapping in (**b**) altered genome fraction and (**c**) mutation count groups. Genes with highest frequency in (**d**) altered genome fraction and (**e**) mutation count groups

To identify potential antigens of PAAD, we first screened for the aberrantly expressed genes and detected 2459 overexpressed genes that likely encode tumor-associated antigens **(**Fig. 1a**)**. A total of 8850 mutated genes potentially encoding for tumor-specific antigens were then filtered by analyzing altered genome fraction and mutation counts in individual samples **(**Fig. 1b and c**)**. Mutational analysis showed that KRAS proto-oncogene GTPase (KRAS) and tumor protein p53 (p53) were the most frequently mutated genes in terms of both altered genome fraction and mutation counts **(**Fig. 1d and e**)**. Of note, in addition to KRAS, p53 and diacylglycerol kinase beta in top 10 candidates with altered genome fractions, anoctamin 1, BCL2-like 2-poly(A) binding protein nuclear 1 readthrough, brain enriched guanylate kinase associated, chromosome 1 open reading frame 87, chromosome 5 open reading frame 34, fem-1 homolog B, as well as immunoglobulin kappa variable 2D-24, all have the same mutation frequency, indicating the potential genomic interaction among them **(**Fig. 1d**)**. High mutation counts were observed in titin, zinc finger protein 814, mucin 16, CUB and Sushi multiple domains 2, SMAD family member 4, dynein axonemal heavy chain 11, phosphatidylinositol-4,5-bisphosphate 3-kinase catalytic subunit alpha, and cyclin dependent kinase inhibitor 2A **(**Fig. 1e**)**. Overall, 926 overexpressed and frequently mutated tumor-specific genes were identified.

### Identification of tumor antigens associated with PAAD prognosis and antigen presenting cells

**Fig. 2:**
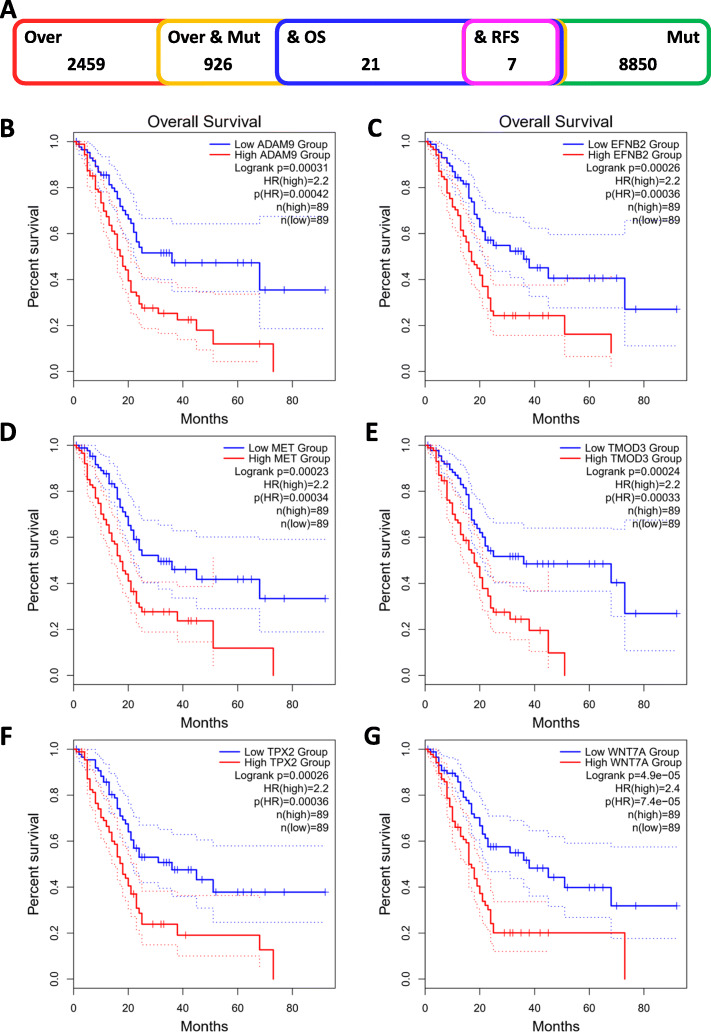
Identification of tumor antigens associated with PAAD prognosis. **a**) Potential tumor antigens (total 926) with high expression and mutation in PAAD, and significant association with OS and RFS (total 7 candidates). **b**-**g** Kaplan-Meier curves showing OS of PAAD patients stratified on the basis of (**b**) ADAM9, (**c**) EFNB2, (**d**) MET, (**e**) TMOD3, (**f**) TPX2 and (**g**) WNT7A expression levels

**Fig. 3:**
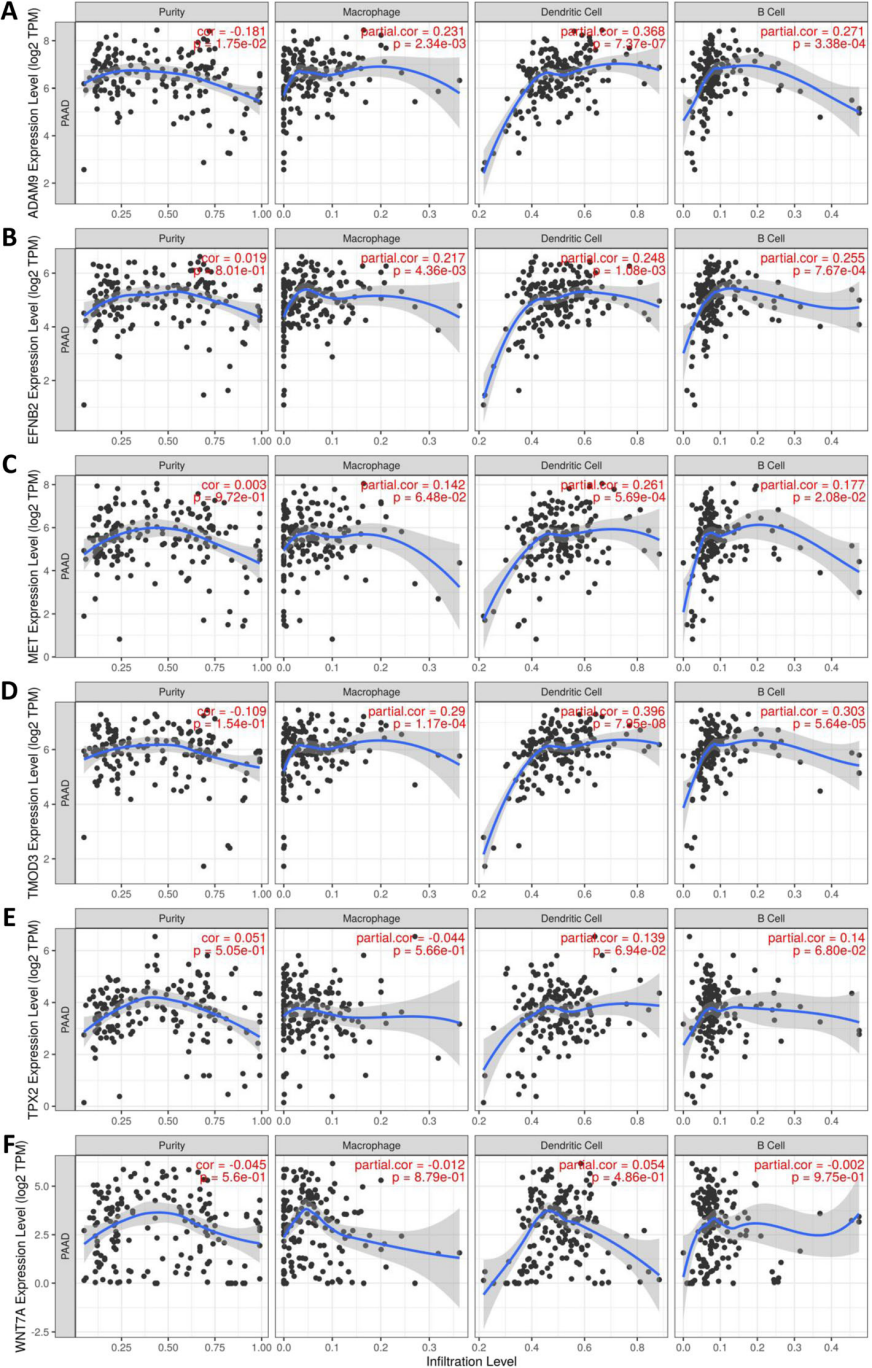
Identification of tumor antigens associated with APCs. Correlation between the expression levels of **a** ADAM9, **b** EFNB2, c MET, **d** TMOD3, **e** TPX2 and ) WNT7A and infiltration of macrophages, dendritic cells and B cells in PAAD tumors

The prognosis-related tumor antigens were next screened from the aforementioned genes as potential candidates for developing mRNA vaccine. Twenty-one genes were closely associated with the OS of PAAD patients, of which 7 genes showed significant correlation with the RFS **(**Fig. 2a**)**. As shown in Fig. 2b, patients overexpressing ADAM metallopeptidase domain 9 (ADAM9) in the tumor tissues had significantly shorter survival compared to the ADAM9^low^ group. Likewise, high expression levels of Ephrin B2 (EFNB2), MET proto-oncogene receptor tyrosine kinase (MET), tropomodulin 3 *Homo sapiens* (TMOD3), TPX2 microtubule nucleation factor (TPX2), wingless-type MMTV integration site family and member 7A (WNT7A) were also associated with poor prognosis **(**Fig. 2c-g**)**. One gene was identified as a favorable prognosis factor (data not shown). Taken together, 6 gene candidates were identified that are critical for PAAD development and progression. Furthermore, higher expression levels of ADAM9, EFNB2, MET, and TMOD3 were significantly associated with increased tumor infiltration of macrophages, DCs and/or B cells **(**Fig. 3a-d**)**. TPX2 and WNT7A also exhibited the upregulated tendency in increased infiltration of immune cells, although their expression levels were more variable **(**Fig. 3e-f**)**. These findings suggest that the identified tumor antigens may be directly processed and presented by the APCs to T cells, and recognized by the B cells to trigger an immune response, and are therefore promising candidates for developing mRNA vaccine against PAAD.

### Identification of potential immune subtypes of PAAD

**Fig. 4:**
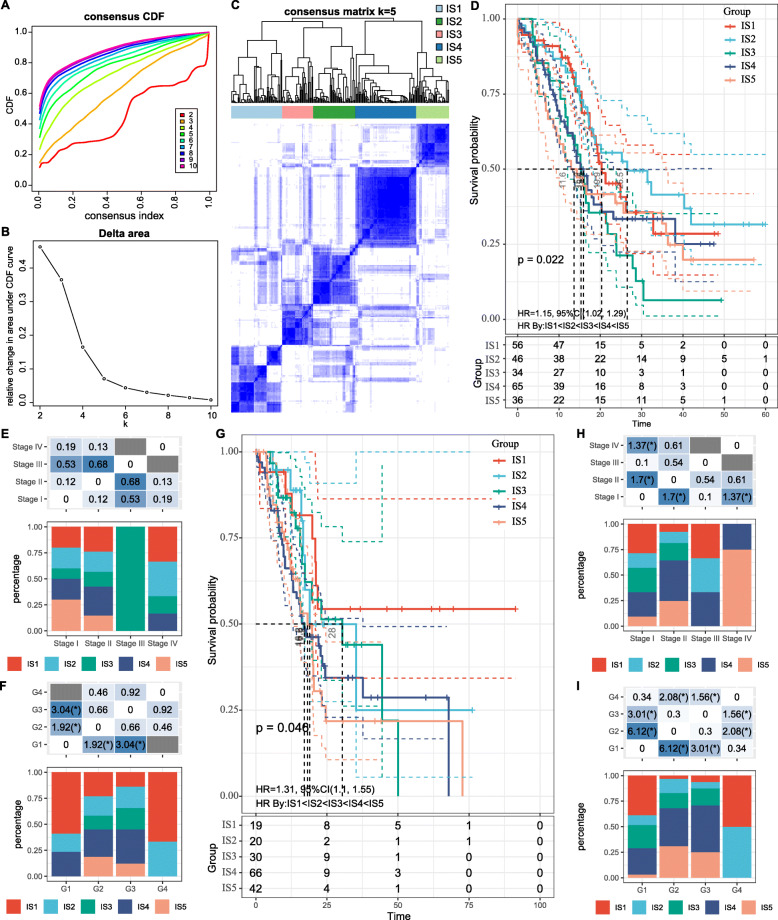
Identification of potential immune subtypes of PAAD. **a** Cumulative distribution function curve and (**b**) delta area of immune-related genes in ICGC cohort. **c** Sample clustering heat map. **d** Kaplan-Meier curves showing OS of PAAD immune subtypes in ICGC cohort. (**e**, **f**) Distribution of IS1-IS5 across PAAD (E) stages and (**f**) grades in ICGC cohort. **g** Kaplan-Meier curves showing OS of PAAD immune subtypes in TCGA cohort. **h**, **i** Distribution ratio of IS1-IS5 across PAAD (**h**) stages and (**i**) grades in TCGA cohort

Immunotyping can be used to mirror the immune status in tumors and their microenvironment, and thus help identify suitable patients for vaccination. Therefore, we analyzed the expression profiles of 1997 immune-related genes in 239 PAAD samples from ICGC database to construct consensus clustering. Based on their cumulative distribution function and function delta area, we chose k = 5 where immune-related genes appeared to be stably clustered **(**Fig. 4a and b**)**, and obtained 5 immune subtypes designated as IS1-IS5 **(**Fig. 4c**)**. IS1 and IS2 were associated with better prognosis, whereas IS3 had the poorest survival probability **(**Fig. 4d**)**. Subtype distribution across different tumor stages and grades indicated that patients diagnosed as differential stage were irregularly clustered **(**Fig. 4e**)**, whereas both grades 1 and 4 were significantly associated with IS1 **(**Fig. 4f**)**. Consistent with the results obtained with the ICGC cohort, the immune subtype was prognostically relevant in TCGA cohort as well **(**Fig. 4g**)**, and significantly altered in different stages **(**Fig. 4h**)**, as well as both grades 1 and 4 showed a great correlation with IS1 **(**Fig. 4i**)**. Taken together, the immunotyping can be used to predict prognosis of PAAD patients and its accuracy is superior to traditional grading and staging, which is consistent across different cohorts.

### The association of immune subtypes with mutational status

**Fig. 5:**
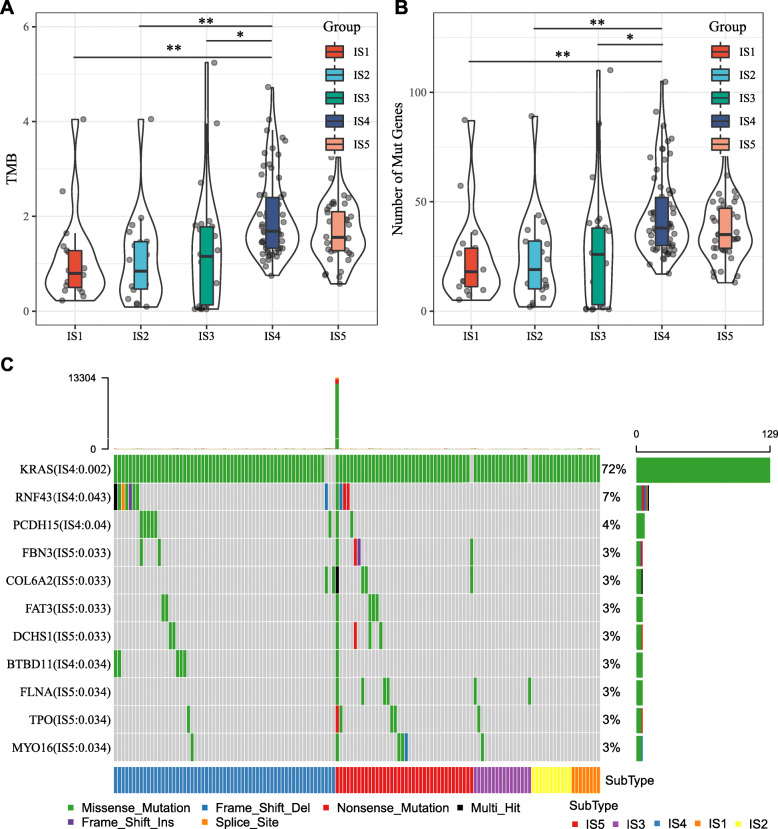
Association between immune subtypes and TMB and mutation. **a** TMB and **b** mutation number in PAAD IS1-IS5. c Eleven highly mutated genes in PAAD immune subtypes. * *p* < 0.05 and ** *p* < 0.01

Higher tumor mutation burden (TMB) and somatic mutation rates are correlated to stronger anti-cancer immunity [28]. Therefore, we calculated the TMB and mutations in each patient using the mutect2-processed mutation dataset of TCGA, and analyzed the same in all immune subtypes. As shown in Fig. 5a, IS4 and IS5 showed significantly higher TMB compared to IS1, IS2 and IS3. Similar trends were seen with the number of mutated genes as well **(**Fig. 5b**)**. Furthermore, 11 genes including KRAS were most frequently mutated in each subtype **(**Fig. 5c**)**. These findings suggest that the immune subtype can predict TMB and somatic mutation rates in PAAD patients, and that patients with IS4 and IS5 may respond positively to the mRNA vaccine.

### Association between immune subtypes of PAAD and immune modulators

**Fig. 6:**
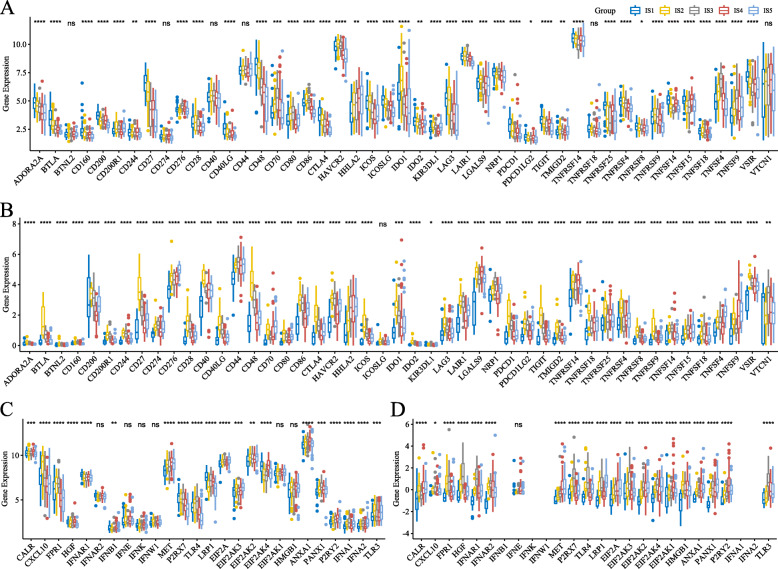
Association between immune subtypes and ICPs and ICD modulators. **a**, **b** Differential expression of ICP genes among the PAAD immune subtypes in (**a**) ICGC and (**b**) TCGA cohorts. **c**, **d** Differential expression of ICD modulator genes among the PAAD immune subtypes in (**c**) ICGC and (**d**) TCGA cohorts. * *p* < 0.01, ** *p* < 0.001, *** *p* < 0.0001, and ****p < 0.00001

Given the importance of immune checkpoints (ICPs) and immunogenic cell death (ICD) modulators in cancer immunity, we next analyzed their expression levels in the different subtypes. Forty-seven ICPs-related genes were detected in both cohorts, of which 41 (87%) genes in ICGC cohort **(**Fig. 6a**)** and 46 (97.9%) in TCGA cohort **(**Fig. 6b**)** were differentially expressed between the immune subtypes. For instance, ADORA2A, BTLA, CD160, CD27, CD40LG, CD48, CTLA4, ICOS, ICOSLG, IDO2, LAG3, LAIR1, NRP1, PDCD1, PDCD1LG2, TIGIT, TNFRSF14, TNFRSF25, TNFRSF4, TNFRSF8, TNFSF14, TNFSF15, TNFSF18, and VSIR were significantly upregulated in IS1 tumors in the ICGC cohort, while ADORA2A, BTLA, CD200, CD200R1, CD244, CD27, CD28, CD40, CD40LG, CD48, CD80, CD86, CTLA4, HAVCR2, ICOS, IDO1, IDO2, LAG3, LAIR1, PDCD1, PDCD1LG2, TIGIT, TMIGD2, TNFRSF18, TNFRSF25, TNFRSF4, TNFRSF8, TNFRSF9, and VSIR were overexpressed in the IS2 tumors in TCGA cohort. Furthermore, the overall expression level of ICPs in the ICGC cohort was higher than that in TCGA cohort. Twenty-eight ICD genes were detected in the ICGC cohort, of which 22 (78.6%) were differentially expressed among the immune subtypes **(**Fig. 6c**)**. Likewise, 25 ICD genes were expressed in TCGA cohort, of which 24 (96%) showed significant differences between the subtypes **(**Fig. 6d**)**. For instance, CALR, IFNB1, MET, EIF2AK4 and P2RY2 were significantly upregulated in IS4 tumors in ICGC cohort, while MET, LRP1, EIF2A, ANXA1 and PANX1 showed significantly higher expression levels in IS5 tumors in TCGA cohort. Therefore, immunotyping can reflect the expression levels of ICPs and ICD modulators and be treated as potential therapeutic biomarkers for mRNA vaccines.

### Association between immune subtypes and tumor markers

**Fig. 7:**
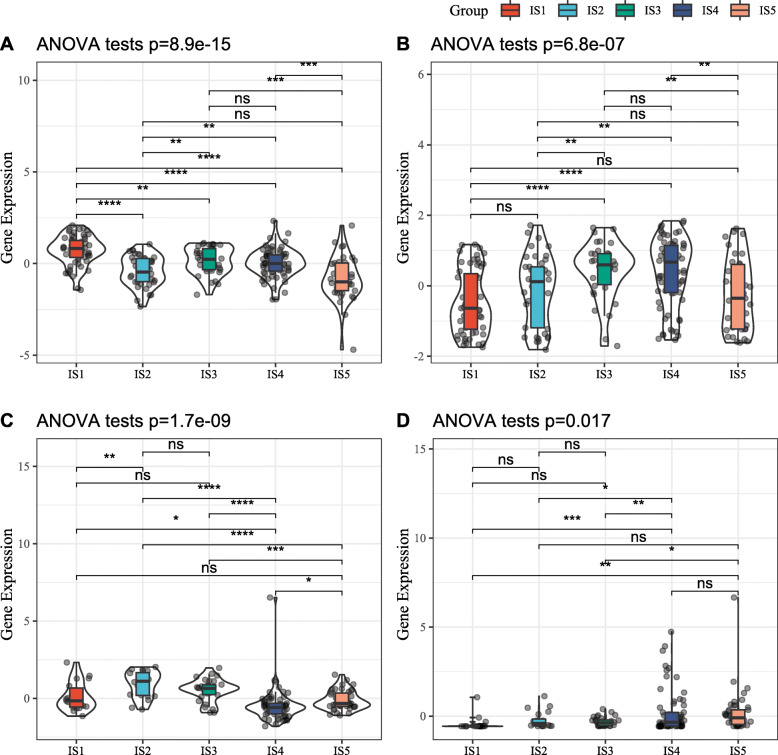
Association between immune subtypes and CA199 and CA125. **a**, **b** CA199 (**a**) and CA125 (**b**) expression in PAAD immune subtypes in ICGC cohorts. **c**, **d** CA199 (**a**) and CA125 (**b**) expression in PAAD immune subtypes in TCGA cohorts

CA125 and CA199 are established prognostic and diagnostic makers of PAAD, and higher levels of both are indicative of cancer progression, poor prognosis or cancer relapse. In this study, both ICGC and TCGA cohorts displayed significant differences in CA199 and CA125 expression levels across the immune subtypes. In the ICGC cohort for example, IS1 as well as IS3 and IS4 showed higher CA199 and CA125 expression respectively **(**Fig. 7a and b**)**, whereas IS2 and IS4 respectively had elevated CA199 and CA125 in TCGA cohort **(**Fig. 7c and d**)**. However, these results contradict the better prognosis observed in the IS1 and IS2 patients. Furthermore, the differential expression of CA199 and not CA125 was relatively consistent in both cohorts, indicating that CA125 levels are highly susceptible to biological or environmental factors. Taken together, immune subtype is superior to CA199 and CA125 in predicting PAAD patient prognosis.

### Cellular and molecular characteristics of immune subtypes

**Fig. 8:**
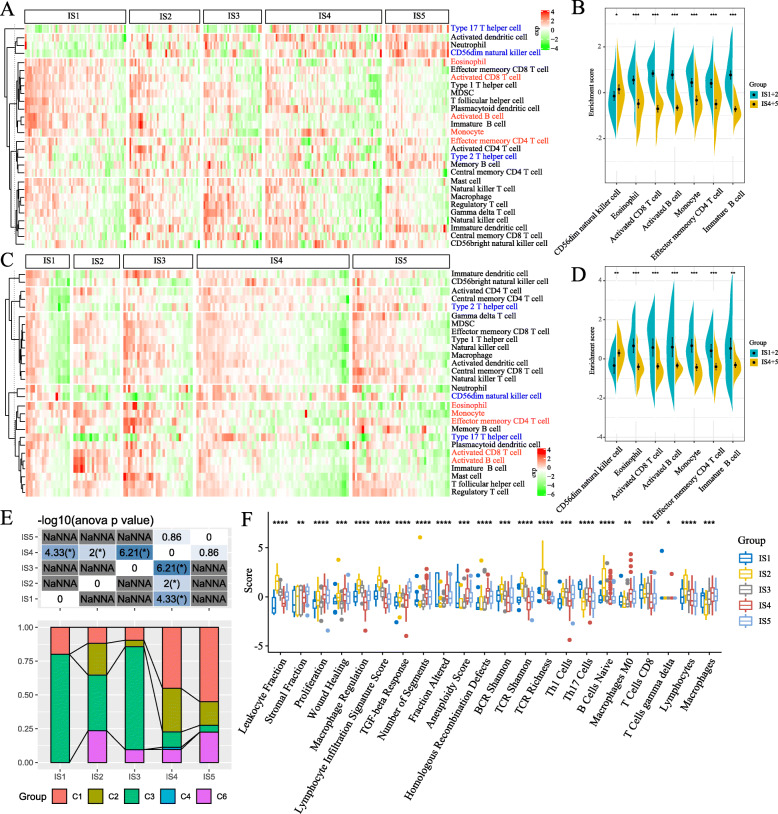
Cellular and molecular characteristics of immune subtypes. **a**, **c** Differential enrichment scores of 28 immune cell signatures among PAAD immune subtypes in (**a**) ICGC and (**c**) TCGA cohorts. **b**, **d** Differential enrichment scores of 7 prognostically relevant immune cell signatures in (**b**) ICGC and (**d**) TCGA cohorts. **e** Overlap of PAAD immune subtypes with 6 pan-cancer immune subtypes. **f** Differential enrichment scores of 56 immune signatures among PAAD immune subtypes and 22 immune signatures with FDR < 0.01

The response to mRNA vaccine depends on the tumor immune status. Hence, we further characterized the immune cell components in the 5 immune subtypes by scoring 28 previously reported signature genes in both TCGA and ICGC cohorts using ssGSEA. As shown in Fig. 8a, the immune cell components were divided into five clusters. IS4 and IS5 showed similar immune cell scores in ICGC cohorts, while similar distribution was observed in IS1 and IS2. Nevertheless, the immune cell composition was significantly different among the subtypes. For instance, the scores of CD56^dim^ natural killer (NK) cells were significantly higher in IS4 and IS5 compared to IS1 and IS2, while that of eosinophils, activated CD8 T cells, activated B cells, monocytes and effector memory CD4 T cells were higher in IS1 and IS2 relative to IS4 and IS5 **(**Fig. 8b**)**. Thus, IS1 and IS2 are immunological “hot” while IS4 and IS5 are immunological “cold” phenotypes. Similar trends were seen in TCGA cohort as well **(**Fig. 8c and d**)**. These results suggest that immune subtype reflects the PAAD immune status, and can identify suitable patients for mRNA vaccination. The mRNA vaccine with these antigens can induce immune infiltration in patients with immunologically “cold” IS4 and IS5 tumors.

To demonstrate the reliability of this immunotyping, we next explored the correlation between the five immune subtypes and previously reported six pan-cancer immune subtypes (C1-C6), of which PAAD was mostly clustered into C1, C2, C3 and C6 [29]. As shown in Fig. 8e, IS1 and IS3 mainly overlapped with C3, IS2 with C2 and C3, IS4 with C1 and C2, and IS5 with C1, C2 and C6. C3 and C6 were associated with better and inferior prognoses respectively, whilst C1 and C2 indicated intermediate prognoses. These results corresponded to prolonged survival observed in patients with IS1 and IS2 tumors compared to IS4 and IS5. Interestingly, IS1 patients with better prognosis and IS3 patients with poorest survival largely overlapped with C3. These findings not only demonstrate the reliability of our immunotyping method but also augment previous classification. We also evaluated the correlation between the immune subtypes and 56 previously defined molecular signatures, and identified 22 significantly associated immune-related signatures with FDR < 0.01 as the threshold. As shown in Fig. 8f, IS2 had the highest scores for lymphocyte infiltration, leukocyte fraction, Th1 cells, B cells naïve and lymphocyte signatures, but scored low for stromal fraction and TGF-β response. Thus, IS2 was associated with an overall favorable immune-activated phenotype and characterized by a highly diverse immune signature. IS1 on the hand was characterized by moderate immune infiltration and highest Th17 cells and CD8^+^ T cells as well as low reactive stroma and TGF-β response signatures. In contrast, the low scores of CD8^+^ T cells, lymphocyte and stromal fraction and TGF-β response signatures in IS4 are indicative of an immunologically cold phenotype. IS5 scored low for CD8^+^ T cells and lymphocytes but had high scores for stromal fraction and TGF-β response, suggesting immune-cold phenotype as well as an immunosuppressive microenvironment. IS3 demonstrated intermediate immune infiltration along with elevated reactive stroma and TGF-β response, which strongly point to an immunosuppressive phenotype. Taken together, the immune subtypes mirror the cellular and molecular signatures in PAAD patients, indicating their immune status. Hence, the immune subtypes are promising biomarkers for mRNA vaccine and patients with immunologically cold” IS4 and IS5 tumors with/out the immunosuppressive microenvironment are potentially suitable candidates for mRNA vaccination.

### Immune landscape of PAAD

**Fig. 9:**
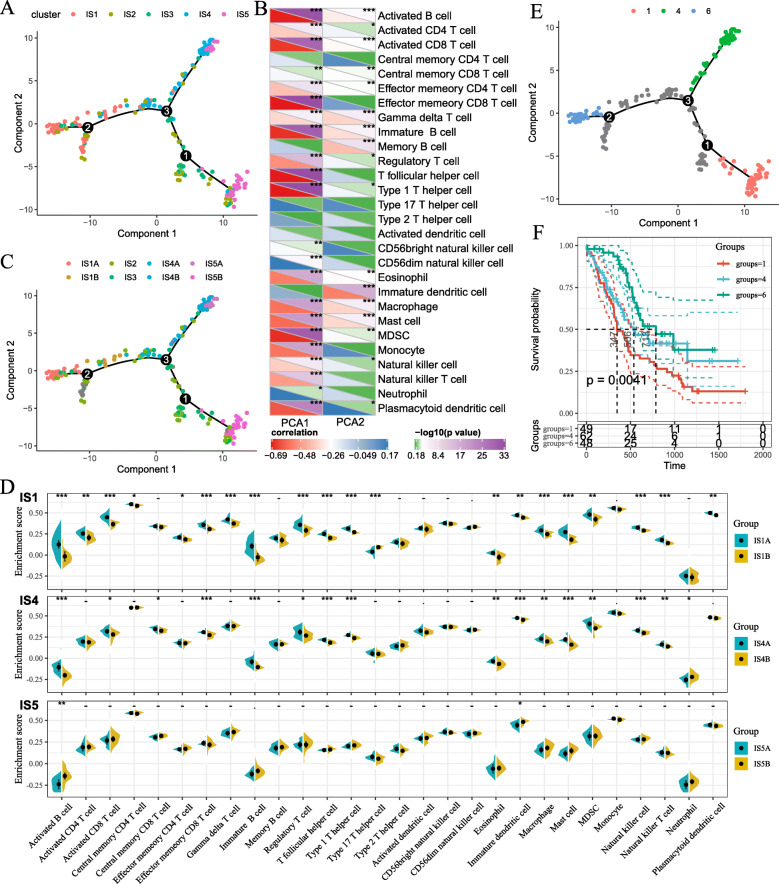
Immune landscape of PAAD. **a** Immune landscape of PAAD. each point represents a patient and the immune subtypes are color-coded. The horizontal axis represents the first principal component and the vertical axis represents the second principal component. **b** Heat map of two principal components with 28 immune cell signatures. **c** Immune landscape of the subsets of PAAD immune subtypes. **d** Differential enrichment scores of 28 immune cell signatures in the above subsets. **e** Immune landscape of samples from three extreme locations and (**f**) their prognostic status

The immune gene expression profiles of individual patients were used to construct the immune landscape of PAAD **(**Fig. 9a**)**. As shown in Fig. 9b, the horizonal axis was correlated to various immune cells, of which effector memory CD8 T cells, type 1 T helper cells and T follicular helper cells showed the most negative correlation, whilst the vertical coordinate was mostly negatively associated with immature DCs. The integral distribution of IS1 was opposite to that of IS4 and IS5. In addition, the same subtype also displayed opposing distribution, indicating significant intra-cluster heterogeneity within subtypes, especially within IS2 and IS3. Based on the location of immune cell populations, IS1, IS4 and IS5 were each further divided into two subsets **(**Fig. 9c**)**, and the enrichment scores of several immune cells were significantly different between subsets **(**Fig. 9d**)**. For example, IS1B showed lower counts of activated B cells, activated CD4^+^ T cells, activated CD8^+^ T cells, effector memory CD8^+^ T cells, regulatory T cells and myeloid-derived suppressor cells (MDSCs), while IS4B scored lower in terms of activated B cells, activated CD8^+^ T cells, effector memory CD8^+^ T cells, regulatory T cells and MDSCs. Thus, the mRNA vaccine may be relatively viable in IS1B and more effective in IS4B. Furthermore, samples with extreme distributional positions in the immune landscape were subjected to prognostic comparison and patients in group 6 showed the best survival probability, which is consistent with aforementioned results **(**Fig. 9e and f**)**. Taken together, the immune landscape based on immune subtypes can precisely identify immune components of each PAAD patients as well as predict their prognoses, which is favorable for selecting personalized therapeutics for mRNA vaccine.

### Identification of immune gene co-expression modules and hub genes of PAAD

**Fig. 10:**
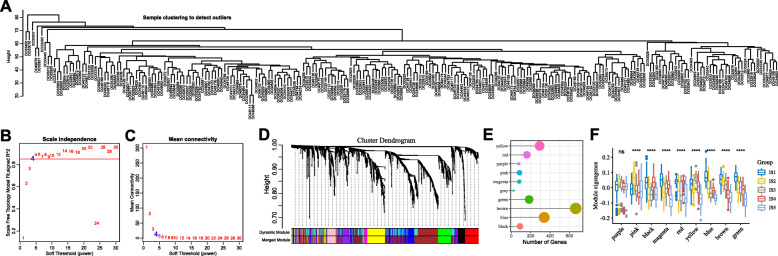
Identification of immune gene co-expression modules of PAAD. **a** Sample clustering. **b** Scale-free fit index for various soft-thresholding powers (β). **c** Mean connectivity for various soft-thresholding powers (**d**) Dendrogram of all differentially expressed genes clustered based on a dissimilarity measure (1-TOM). **e** Gene numbers in each module. **f** Differential distribution of feature vectors of each module in PAAD subtypes

**Fig. 11:**
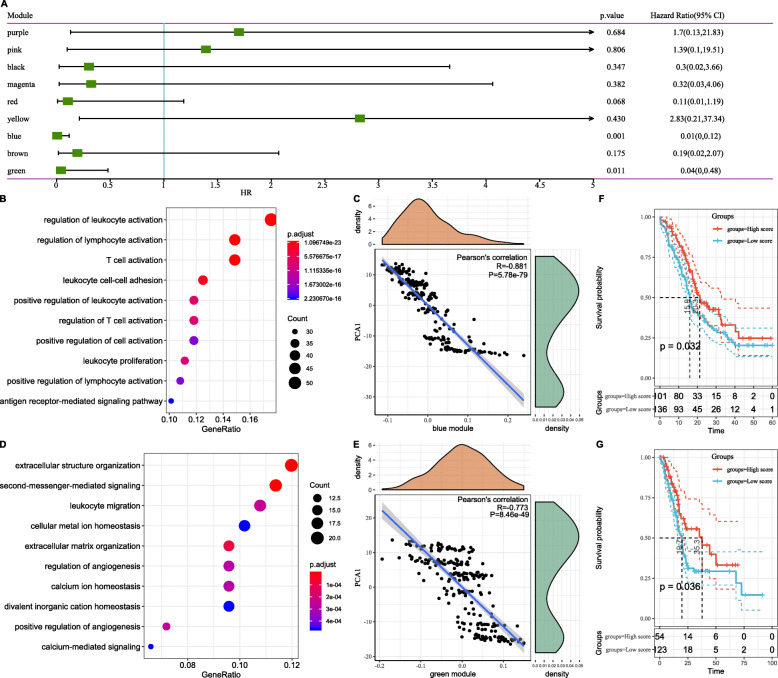
Identification of immune hub genes of PAAD. **a** Forest maps of single factor survival analysis of 11 modules of PAAD. **b** Dot plot showing top 10 KEGG terms in the blue module. The dot size and color intensity represent the gene count and enrichment level respectively. **c** Correlation between blue module feature vector and second principal component in immune landscape. **d** Dot plot showing enriched terms in green module. **e** Correlation between green module feature vector and second principal component in immune landscape. **f** Differential prognosis in blue module with high and low mean. **g** Differential prognosis in green module with high and low mean

Immune gene co-expression modules were identified by clustering the samples by WGCNA **(**Fig. 10a**)** with a soft threshold of 4 for scale-free network **(**Fig. 10b and c**)**. The representation matrix was then converted to an adjacency and then to a topological matrix. We used the average-linkage hierarchy clustering method with minimum 30 genes for each network following the standard of hybrid dynamic shear tree. Eigengenes of each module were calculated and the close modules were merged into a new one with height = 0.25, deep split = 4 and min module size = 30. As shown in Fig. 10d, we obtained 10 co-expression modules with 1997 transcripts, of which the genes in grey module did not cluster with the rest **(**Fig. 10e**)**. We further analyzed the distribution of 5 immune subtypes in the eigengenes of 9 (except grey) modules, and detected significantly different distribution in 8 modules **(**Fig. 10f**)**. IS5 showed the lowest eigengenes in magenta, red, yellow, blue, brown and green modules and IS1 showed the highest eigengenes in the black, magenta, red, blue, brown and green modules. Thus, IS5 corresponded to immunologically cold and IS1 to inflamed tumors. Further prognostic correlation analysis showed that the blue and green modules were significantly associated with the prognosis of PAAD (Fig. 11a). Moreover, the blue module was related to T cell activation but was negatively associated with the component 1 of immune landscape **(**Fig. 11b and c**)**. Likewise, the green module related to leukocyte migration showed consistent negative correlation as well **(**Fig. 11d and e**)**. Analysis of prognostically relevant genes of the blue module showed that higher expression scores correlated with better prognosis in the ICGC and TCGA cohorts, which is consistent with abovementioned findings **(**Fig. 11f and g**)**. The infiltration and activation of T cells and other immune cells in tumor tissues, as well as inhibition of immune-suppressive cells, largely determine the therapeutic potential of mRNA vaccine in cancer patients with specific immune subtype. Accordingly, mRNA vaccine might be not suitable to patients with high expression of genes clustered into blue and green modules. Finally, three hub genes with > 90% relevance in blue module were identified, including MAP 4 K1, TBC1D10C and TRAF3IP3, which are potential biomarkers for mRNA vaccine.

## Discussion

To the best of our knowledge, this is the first study to screen for PAAD antigens for developing mRNA vaccine. We constructed the aberrantly expressed and mutational landscape of PAAD and identified a series of targetable antigens, of which ADAM9, EFNB2, MET, TPX2, TMOD3 and WNT7A are promising mRNA vaccine candidates. Their upregulation was not only associated with poor prognosis and RFS, but also high APC and B cell infiltration. Therefore, these antigens play critical roles in the development and progression of PAAD and can be directly processed and presented to CD8^+^ T cells in the event of adequate lymphocyte infiltration to induce an immune attack. Although these candidates have to be functionally validated, their potential for mRNA development is supported by previous reports. For example, ADAM9 is involved in tumor cell metastasis, proliferation, angiogenesis and immune evasion [30, 31] and high levels of ADAM-9 in pancreatic cancer is associated with short OS and higher tumor grade and progression [32, 33]. Furthermore, ADAM9 is upregulated by aberrant KRAS/NF-kB signaling in cancer cells, and knocking down ADAM9 suppresses KRAS and MEK-ERK signaling [34, 35]. EFNB2 acts as a oncogene in pancreatic ductal adenocarcinoma (PDAC) and is markedly upregulated in PDAC and correlated to clinical stage and Ki67 expression levels in PDAC [36, 37]. EFNB2 knockout suppresses cancer cell proliferation, migration and invasion by inducing p53/p21-mediated G0/G1 phase blockade [37, 38]. The aberrantly high levels and activation of MET are closely related with cancer onset, progression and metastasis [39] and its upregulated in pancreatic cancer associated with grade [40]. Pharmacological or genetic ablation of MET significantly prolonged survival of mice bearing PDAC xenografts. TPX2 is a prognostic biomarker of KARS-mutant PDAC [41, 42], and its high expression levels correlate to shorter survival and increased frequency of oncogenic KRAS mutations [41]. Targeted inhibition of KRAS decreased TPX2 expression in PDAC cells, which in turn reduced clonogenic and anchorage-independent growth and migration *in vitro*^41^. TMOD3 is a tumor suppressor target and an independent prognostic marker of PDAC [43], and its overexpression in hepatocellular carcinoma correlates with tumor progression and poor patient survival, and TMOD3-knockout liver cancer cells have decreased proliferation, invasion and migration [44, 45]. WNT7A is a ligand of the Wnt/β-catenin signaling pathway, and highly expressed in PDAC wherein it positively correlates with poor prognosis and lymph node metastasis. Inhibition of WNT7A disrupts Wnt/β-catenin signaling in pancreatic cancer cell lines and suppresses proliferation [39].

Given that mRNA vaccine is only beneficial for a fraction of cancer patients, we classified PAAD into five immune subtypes based on immune gene expression profiles for selecting the appropriate population for vaccination. The five immune subtypes exhibited distinct molecular, cellular and clinical characteristics. Patients with IS1 and IS2 tumors showed better prognosis compared to other subtypes in both ICGC and TCGA cohorts. This suggests the immunotyping can be used for predicting the prognoses of PAAD patients, and we demonstrated its superior predictive accuracy compared to established tumor makers like CA199 and CA125, as well as traditional staging and grading. In addition to prognostic prediction, immunotyping is also indicative of the therapeutic response to mRNA vaccine. For instance, patients with IS4 and IS5 tumors with higher TMB and somatic mutation rates may have greater responsiveness to mRNA vaccine. The high expressions of ICPs in IS1 tumors in ICGC cohort, and in IS2 tumors in TCGA cohort suggest an immunosuppressive tumor microenvironment, which may inhibit the mRNA vaccine from eliciting an effective immune response. In contrast, the elevated expression of ICD modulators in IS4 tumors in ICGC cohort, and in IS5 tumors in TCGA cohort are suggestive of greater potential of mRNA vaccine in these immune subtypes. Interestingly, MET is also an ICD modulator, and may therefore be a stronger candidate for mRNA vaccine compared to the other selected antigens. Furthermore, the complex immune landscape of PAAD indicates considerable heterogeneity between individual patients as well as within the same immune subtype, which narrows down the immune components for developing personalized mRNA vaccine-based therapeutics. MAP 4 K1, TBC1D10C and TRAF3IP3 were identified as hub genes in the blue module, and their upregulation was negatively associated with the component 1 of immune landscape, suggesting that patients expressing high levels of these genes may not respond to the mRNA vaccine.

Since the tumor immune status is a determinant of mRNA vaccine efficacy, we further characterized the immune cell components in the different subtypes. IS1 and IS2 showed significantly elevated scores of eosinophils, activated CD8 T cells, activated B cells, monocytes and effector memory CD4 T cells compared to IS4 and IS5, while CD56dim NK cells scored higher in IS4 and IS5. This indicated that IS1 and IS2 are immunological “hot”, and IS4 and IS5 are immunological “cold” phenotypes. The molecular signatures of these tumors were consistent with the immune signatures, indicating that patients with different immune subtypes respond distinctly to mRNA vaccine. For instance, IS4 was associated with low expression of CD8^+^ T cells, lymphocyte and stromal fraction and TGF-β response gene signatures, indicating an immunologically cold phenotype. To circumvent poor immunogenicity of these tumors, mRNA vaccines that stimulate the immune system by triggering immune cell infiltrating may be a suitable option. For the immune-cold and immunosuppressive IS5 tumors exhibiting low CD8^+^ T cells the least lymphocyte and high stromal fraction and TGF-β signatures, combining the vaccine with ICB or ICD modulators may reinvigorate the immune system and increase immune cell infiltration. IS3 demonstrated moderate immune infiltration and immunosuppressive phenotype with elevated reactive stroma and TGF-β response signatures, and may respond to ICB or other strategies. IS1 and IS2 exhibited a favorable immune-activated phenotype with low stromal fraction and TGF-β response gene expression, and may therefore be responsive to ICB.

Based on previous immunotyping studies, PAAD was classified into the C1-C6 subtypes. Most patients were clustered into the C1, C2, C3 and C6 subtypes. C3 is associated with superior, C1 and C2 with moderate, and C6 with inferior prognoses. In this study, PAAD was differentiated into IS1-IS5 subtypes. IS1 and IS3 mainly overlapped with C3, IS2 with C2 and C3, IS4 with C1 and C2, and IS5 with C1, C2 and C6. These results were in agreement with better survival probability of IS1 and IS2, and the relatively poor prognoses of IS4 and IS5. Interestingly, both IS1 with superior prognosis and IS3 with lowest survival largely overlapped with C3. Therefore, our immunotyping method is reliable and complements the previous classification. Nevertheless, the vaccine antigens and other prognostic markers identified in this study will have to be validated in future studies.

## Conclusions

ADAM9, EFNB2, MET, TMOD3, TPX2 and WNT7A are potential PAAD antigens for mRNA vaccine development. Patients with immune subtypes 4 and 5 are suitable candidates for vaccination. Our findings provide a theoretical basis for developing anti-PAAD mRNA vaccine, predicting patient prognosis and selecting patients for vaccination.

## Data Availability

All data generated and described in this article are available from the corresponding web servers, and are freely available to any scientist wishing to use them for noncommercial purposes, without breaching participant confidentiality. Further information is available from the corresponding author on reasonable request.

## Abbreviations

PAAD: Pancreatic adenocarcinoma
TIME: Tumor immune microenvironment
OS: Overall survival
RFS: Relapse-free survival
IGP: In-group-proportion
ICPs: Immune checkpoints
ICD: Immunogenic cell death
DCs: Dendritic cells
APC: Antigen presenting cells
TIICs: Tumor infiltrating immune cells
KO: Knockout
ICB: Immune checkpoint blockage
TMB: Tumor mutation burden
CA125: Carbohydrate antigen 125
CA199: Carbohydrate antigen 199
ICGC: International Cancer Genome Consortium
PDAC: Pancreatic ductal adenocarcinoma
TCGA: The Cancer Genome Atlas
ssGSEA: Single-sample GSEA
KRAS: KRAS proto-oncogene GTPase
p53: Tumor protein p53
EFNB2: Ephrin B2
MET: MET proto-oncogene receptor tyrosine kinase
TMOD3: Tropomodulin 3 *Homo sapiens*
TPX2: TPX2 microtubule nucleation factor
WNT7A: Wingless-type MMTV integration site family member 7A
MDSCs: Myeloid-derived suppressor cells
